# Artificial Intelligence in Postoperative Care: Assessing Large Language Models for Patient Recommendations in Plastic Surgery

**DOI:** 10.3390/healthcare12111083

**Published:** 2024-05-24

**Authors:** Cesar A. Gomez-Cabello, Sahar Borna, Sophia M. Pressman, Syed Ali Haider, Ajai Sehgal, Bradley C. Leibovich, Antonio J. Forte

**Affiliations:** 1 Division of Plastic Surgery, Mayo Clinic, Jacksonville, FL 32224, USA; 2 Center for Digital Health, Mayo Clinic, Rochester, MN 55905, USA; 3 Department of Urology, Mayo Clinic, Rochester, MN 55905, USA

**Keywords:** large language models, artificial intelligence, plastic surgery, postoperative care, patient resource, patient-centered outcomes, patient satisfaction

## Abstract

Since their release, the medical community has been actively exploring large language models’ (LLMs) capabilities, which show promise in providing accurate medical knowledge. One potential application is as a patient resource. This study analyzes and compares the ability of the currently available LLMs, ChatGPT-3.5, GPT-4, and Gemini, to provide postoperative care recommendations to plastic surgery patients. We presented each model with 32 questions addressing common patient concerns after surgical cosmetic procedures and evaluated the medical accuracy, readability, understandability, and actionability of the models’ responses. The three LLMs provided equally accurate information, with GPT-3.5 averaging the highest on the Likert scale (LS) (4.18 ± 0.93) (*p* = 0.849), while Gemini provided significantly more readable (*p* = 0.001) and understandable responses (*p* = 0.014; *p* = 0.001). There was no difference in the actionability of the models’ responses (*p* = 0.830). Although LLMs have shown their potential as adjunctive tools in postoperative patient care, further refinement and research are imperative to enable their evolution into comprehensive standalone resources.

## 1. Introduction

The advancement of artificial intelligence (AI) offers new opportunities for healthcare improvements and individualized patient care. Large language models (LLMs) represent a breakthrough in applied AI and medical practice. They learn and understand the complex patterns and structures in typical language by leveraging natural language processing (NLP) and deep learning (DL) techniques, in particular, transformer architectures [1]. LLMs can process, interpret, and summarize vast amounts of internet data in real time and generate human-like text responses [2,3]. Since their release, LLMs have shown promise in providing accurate medical knowledge throughout distinct medical fields, encouraging the medical community to actively explore their potential applications and determine how to best leverage their capabilities.

Plastic surgery is a constantly innovating field that relies on updated, accurate tools to provide patient-centered outcomes [4]. In particular, cosmetic surgery is a major ever-evolving field with a rising demand due to its growing social acceptance. In 2022, there were 26.2 million surgical and minimally invasive cosmetic and reconstructive procedures in the United States, with a 19% increase in cosmetic surgery since 2019 [5]. Plastic surgeons are challenged to provide comprehensive information and support to an increasing number of patients [3]. LLMs, in particular, Open AI’s ChatGPT, have proved to be helpful throughout the specialty, with the ability to generate and detect areas for improvement and potential research [4]. ChatGPT has even proved to have a comparable level of knowledge to 50% of first-year integrated plastic surgery residents, as shown by in-service exam scores [6]. 

While most LLM research focuses on their clinical decision capabilities, such as diagnosis and treatment, one very promising application is as a patient resource. Given its accessibility, patients already rely on the Internet as their primary source of information and make decisions based on the information they find, risking themselves being misinformed [2,7]. Previous studies have shown that despite some inherent limitations, LLMs can provide accurate information for patients seeking insights into cosmetic procedure details, outcomes, risks, and benefits [2,3,8]. Additionally, they offer immediate, multilingual, round-the-clock access to information, which is crucial for addressing concerns and questions outside of regular office hours and ensuring equity. 

In plastic surgery, the postoperative period is as important as the surgical procedure itself, and in some cases even more so, as the patient’s final outcome and the procedure’s long-term success depend on it [9]. Although surgeons offer clear verbal and printed postoperative care recommendations, patients may struggle to retain or implement them due to their postoperative emotional state. This forces them to keep looking for their surgeons and their staff’s help outside of office hours, sometimes overburdening the surgical staff. Approaches to decrease these burdens may include limiting notifications or delegating responses to less-trained support staff [10]. These limit access to high-quality care and lead to the common patient misconception that they are forgotten once their surgery has finished. The immediate and accurate responses provided by LLMs can reduce anxiety and improve patient satisfaction [11,12,13].

Building on previous work assessing LLMs’ use in the perioperative period [2,3,8,14,15], we aimed to evaluate and compare the ability of the currently readily available LLMs, ChatGPT-3.5, ChatGPT-4, and Google’s Gemini, formerly Bard, to provide postoperative care recommendations to patients who underwent any one of the five most common cosmetic plastic surgery procedures according to the American Society of Plastic Surgeons (ASPS) in 2022 [5] in the absence of utilizing a retrieval-augmented generation (RAG) approach. As accessibility is fundamental to achieving equal health distribution, we decided to include the publicly available version of ChatGPT in our comparison. 

## 2. Methods

### 2.1. Study Design 

**Figure 1:**
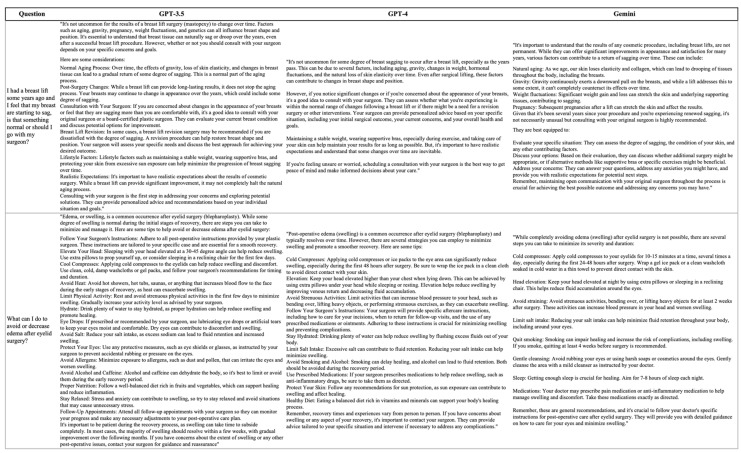

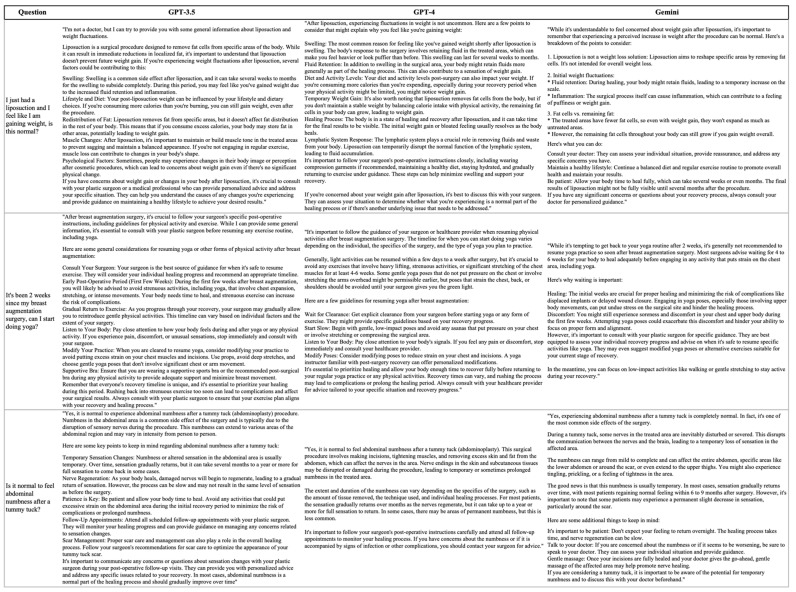
Examples of the questions provided to the LLMs.

We created a set of 32 questions addressing the most common concerns patients have at our clinic after liposuction (n = 7), breast augmentation (n = 6), abdominoplasty (n = 6), mastopexy (n = 6), and blepharoplasty (n = 7). Each question was presented to each LLM using a combination of specialized medical terminology and common language to reflect the various ways in which a typical plastic surgery patient might phrase their questions. Moreover, we did not employ any specialized prompting engineering techniques, such as contextualization or role play, to ensure that the responses obtained were not a result of these methods, as a typical patient may not be aware of them or be comfortably familiarized with them. We only asked each question once and in different chats. After every question was asked to one model, another was tested. Figure 1 portrays an example of the questions. In addition, we have included the full set of questions provided to the LLMs and the responses retrieved in Supplementary File S1 and in a Supplementary File S2, respectively.

### 2.2. Employment of Language Models 

Our goal was to evaluate the capabilities of the currently available LLMs to the public without utilizing an RAG approach. To do this, we used the models’ responses based on their current training. After learning grammar, vocabulary, and context during pre-training with a vast dataset of internet text, the models are fine-tuned on specific datasets tailored to specialized tasks such as text generation or conversation [1]. Models such as GPT and Gemini are further trained using extensive internet-sourced text data, such as books, articles, wikis, and websites, including high-level-evidence medical research [1,14,16]. This training enables the models to provide accurate medical information. 

### 2.3. Evaluation Tools 

We evaluated and compared each LLM response’s accuracy, readability, understandability, and actionability. 

For medical accuracy, we utilized a 5-point Likert scale with the following values: 1 point: completely incorrect, 2 points: partially incorrect, 3 points: partially correct and incorrect, 4 points: partially correct, and 5 points: completely correct. To score each answer, we used as ground truth the ASPS’s webpage [17,18,19,20,21] and textbooks such as *The Art of Aesthetic Surgery* [22] and *Essentials of Plastic Surgery* [23]. Three independent authors (C.A.G.C., S.B., and S.A.H.) analyzed and graded the responses. Any discrepancies were discussed to reach a consensus, and when not possible, the most common score assigned by the authors was used.

We used the Flesch Reading Ease (FRE) score and the Flesch–Kincaid Grade Level (FKGL) to assess readability. The FRE gives a score between 1 and 100, with scores around 100 meaning that the document is extremely easy to read. Scoring between 70 and 80 is equivalent to school-grade level 8. The FKGL assesses the approximate reading grade level of a text. If a text has an FKGL of 8, the reader needs a grade 8 reading level or above to understand it. Both tests take into account the number of sentences, words, and syllables to emit a score [24]. According to the American Medical Association (AMA) [25] and the National Institute of Health (NIH) [26], readability scores should not exceed 6th and 8th-grade levels, respectively. We calculated the FRE score and the FKGL for every model’s response using a free online calculator.

We employed the Patient Education Materials Assessment Tool (PEMAT) [27] to measure the understandability and actionability of the LLMs’ responses. While it is recommended for the evaluation of printed or audiovisual materials, we decided to use it as it is a systematic method designed to determine whether patients will be able to understand and act on the information provided. According to the developers’ website, materials are understandable when consumers can process and explain key messages regardless of their backgrounds and level of health literacy. On the other hand, materials are actionable when patients can identify what they can do based solely on the information provided, regardless of their background and health literacy level. There are two versions of the PEMAT, for printable and for audiovisual materials. We used the printable version, which consists of 17 understandability items and 7 actionability items. Each item was rated as agree (1 point), disagree (0 points), or N/A (not applicable). The sum of the total points was divided by the total possible points (excluding items with N/A), and the result was multiplied by 100. A higher score indicates that the material is more understandable or actionable. We gave an understandability and actionability score to every response given by the LLMs. 

### 2.4. Statistical Analysis 

We calculated and charted the mean, mode, standard deviation (SD), and range of the evaluated metrics of the models’ responses using a Microsoft Excel spreadsheet (Version 2403 Build 16.0.17425.20236) 64-bit). To compare the models’ performance, we employed the analyses of variance (ANOVA) and Tukey’s post hoc analysis when applicable. ANOVA and Tukey’s post hoc test were calculated using Microsoft Excel’s statistical package. We considered a *p*-value < 0.05 as statistically significant. 

## 3. Results 

### 3.1. Medical Accuracy 

**Figure 2:**
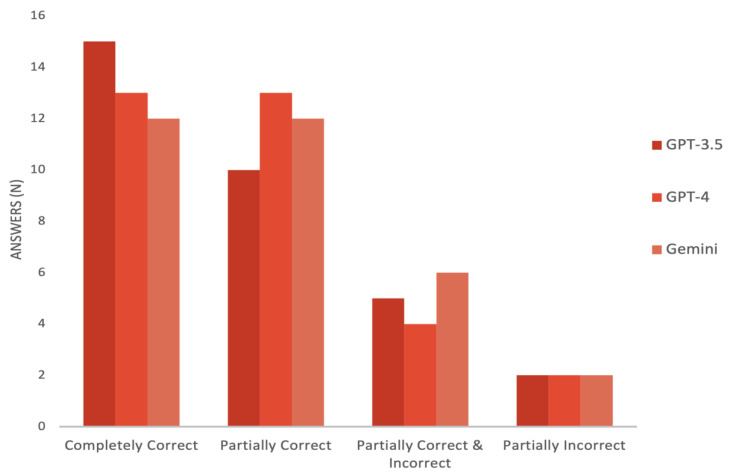
Likert scale scores per LLM.

Overall, the three LLMs provided accurate information, with no statistically significant difference (*p* = 0.85). ChatGPT-3.5 obtained the highest mean score of 4.19 ± 0.93, followed by GPT-4 with a mean of 4.16 ± 0.88 and Gemini with a mean of 4.06 ± 0.91. The three models’ scores ranged between 2 and 5 points, with 81% of the answers provided by ChatGPT-4 scoring higher than 4. The same was true for 78% and 75% of ChatGPT-3.5’s and Gemini’s answers, respectively (Figure 2).

### 3.2. Readability 

**Figure 3:**
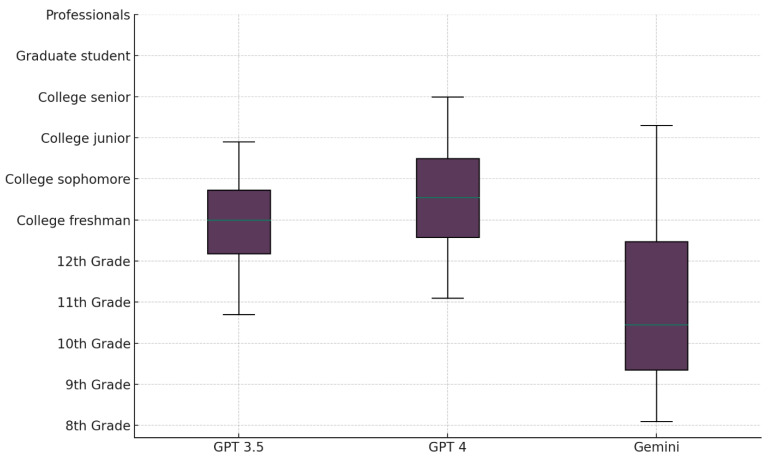
A box and whisker plot of the readability scores per LLM. Each box represents the interquartile range (25–75% of data), the line inside the box shows the median, and the whiskers extend to the smallest and largest values within 1.5 times the interquartile range from the lower and upper quartiles, respectively.

Gemini’s responses were significantly more readable than those of ChatGPT-3.5 and 4. The average FRE score for Gemini was 43.72 ± 10.2, which was significantly higher than ChatGPT-3.5’s 33.7 ± 6.8 and ChatGPT-4’s 33.7 ± 6.2 (*p* = 0.001). This translated to a significantly lower FKGL average for Gemini’s responses (10.92 ± 2.0) than those of ChatGPT-3.5 (12.88 ± 1.0) and ChatGPT-4 (13.6 ± 1.3), with a *p*-value of 0.001. While 53% of the responses provided by Gemini required a college reading level, it was the only model with responses requiring a 10th to 12th-grade reading level (31%). In contrast, almost 72% of ChatGPT-4 responses required a college reading level, and 41% of those from ChatGPT-3.5 were nearly at a college graduate reading level (Figure 3). 

### 3.3. Understandability and Actionability 

**Figure 4:**
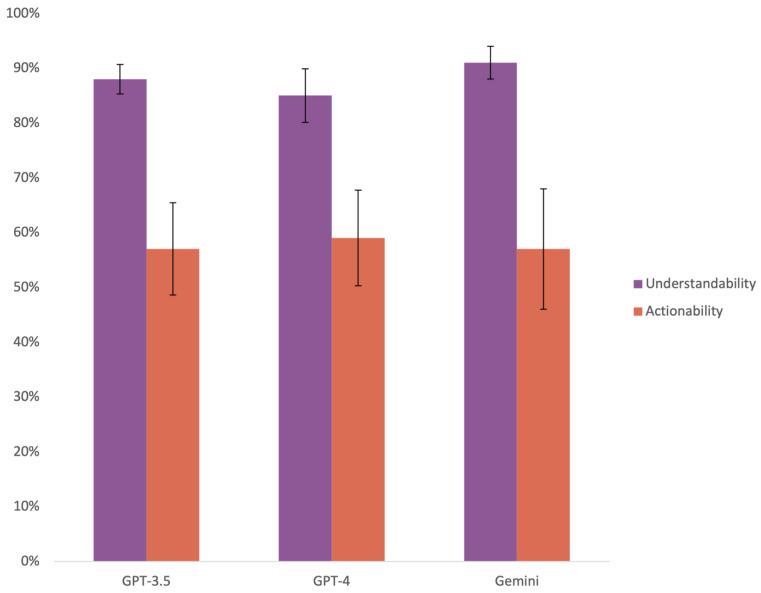
Understandability and actionability scores per LLM. Bars represent average score; error bars represent standard deviation.

Gemini provided more understandable responses, with an average PEMAT understandability score of 90.97 ± 3.0%. This was statistically different from ChatGPT-4, with an average score of 85.13 ± 4.9% (*p* = 0.001), and ChatGPT-3.5, which averaged at 88.31 ± 2.7% (*p* = 0.014). ChatGPT-3.5 also obtained a significantly higher average understandability score than GPT-4, with a *p*-value of 0.002. Regarding actionability, ChatGPT-4 provided the most actionable responses, with an average of 58.7 ± 8.7%. Both ChatGPT-3.5 and Gemini averaged 57.5%; however, Gemini’s responses ranged among higher scores, 40–80%, as compared to ChatGPT-3.5, which scored as lower as 20% and only reached a maximum score of 60%. Nevertheless, there was no statistically significant difference in actionability among these models (*p* = 0.83) (Figure 4). 

## 4. Discussion 

In this modern era, the vast, instantaneous access to medical information acts as a double-edged sword. While patients can easily access helpful information to guide their decisions, they can also easily be misinformed, risking making prejudicial decisions. As they are publicly available, LLMs may present as a solution by offering accurate information presented as a human-like conversation text. One study even showed that patients preferred chatbot responses over physicians’ as they were perceived as more empathetic [10]. In specialties with rising demands, such as plastic surgery, these models may be pivotal as they can serve a vast number of patients simultaneously. 

**Figure 5:**
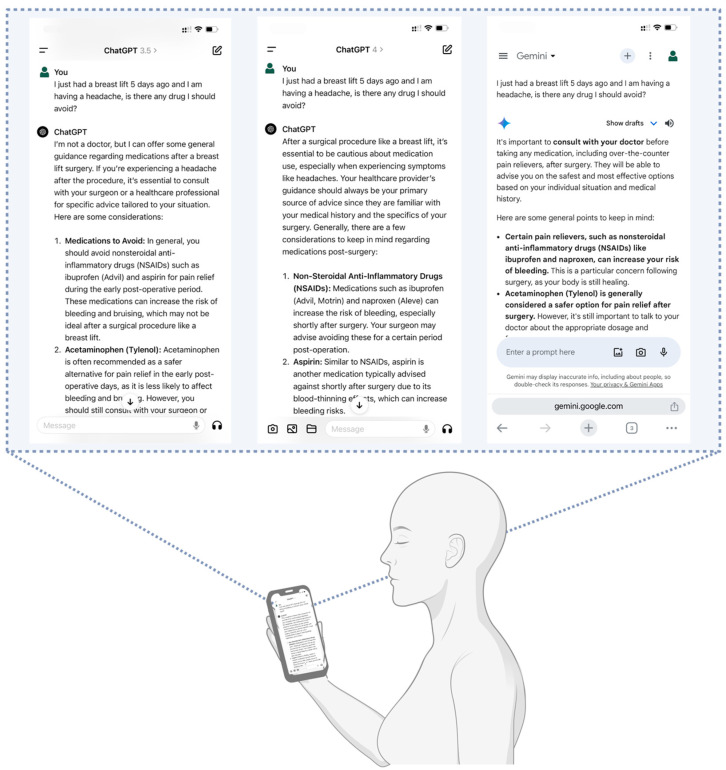
Examples of prompts given to the LLMs and an illustration of the LLMs as adjunctive tools for postoperative care. Created with BioRender [29].

In plastic surgery, several studies have analyzed LLMs’ capabilities for answering patient questions in the pre- and postoperative period for breast surgery [2,3,8], blepharoplasty [14], rhinoplasty [15], and oculoplastic surgery [28]. This underscores their potential as extremely helpful and valuable adjunctive tools for patient management and the importance of exploring their capabilities, with further visualization toward independent tools (Figure 5). To our knowledge, this is the first study comparing the two versions of ChatGPT and Gemini. 

Optimally, LLMs would be able to provide accurate medical information and, when not, at least provide non-harmful advice without over-alerting patients, which would further burden physicians. In our study, the three LLMs provided accurate information at least 75% of the time. Although ChatGPT-3.5 had entirely correct answers almost 47% of the time, it also responded partially incorrectly nearly 22% of the time. The latter was also true for 25% of Gemini’s responses. Conversely, ChatGPT-4 was at least partially correct 81% of the time. However, there was no significant difference in the accuracy among the models. This was the opposite in Al-Sharif et al. [28] and in Abi-Rafeh et al. [2], where GPT-3.5 outperformed Bard in providing comprehensive, accurate responses.

Even though there was no statistical difference, we identified that ChatGPT-4’s responses were more comprehensive and straightforward than those of ChatGPT-3.5 and Gemini, as they were usually preceded or followed by unnecessary, unuseful content. It was common for all of the models to recommend asking or visiting their surgeon, even when the questions were unrelated to life-threatening scenarios. However, Gemini stated four times that it was unable to provide any medical advice as it was just an LLM and instead encouraged patients to visit a doctor. Similarly, ChatGPT-3.5 started its response by saying it was not a doctor before providing an accurate response twice. This may be an attempt to avoid accountability or a way to express their limitations as LLMs, but it was not the case for ChatGPT-4. 

Despite their impressive ability to process information, LLMs still struggle to provide completely accurate responses, which remains the most prevailing concern in their use among specialties [30]. ChatGPT-3.5’s latest update was in March 2022, which hinders its ability to answer questions with updated information after that date. Although ChatGPT-4 was last updated in April 2023, the same can still be true. Both GPT-4 and Gemini can access the Internet to provide updated information; nevertheless, their responses are primarily based on their training data [4]. This risks the models being biased as they cannot only inherit but also amplify biases present in their training data [31,32], perpetuating inequalities related to factors such as race, gender, and socioeconomic status [1,11]. Moreover, LLMs may generate fabricated responses, often referred to as hallucinations, when lacking information, which can result in deviations from established practices [30]. Statistical parity ensures that the demographics used for training the models are the same as the demographics of the population where they will be implemented, which can be achieved with specialized training [31,32]. However, for now, it is crucial to analyze inaccurate responses and verify the information generated by the models [30,31,32].

Our study determined none of the responses as completely incorrect, only partially incorrect. This may be seen in the scenario of an acute postoperative period of a patient who underwent an abdominoplasty and wanted to know how many days she had to stay in bed. ChatGPT-4 suggested gentle walks around the house and no strenuous activities but failed to mention the number of days. On the other hand, Gemini accurately mentioned that it was not recommended to stay in bed as early ambulation was crucial for recovery. However, it did not say why and then contradicted itself by recommending 2 days of strict bed rest followed by short walks and light activity. While low Likert scores do not necessarily mean that the responses might be harmful to the patient, encourage risky behaviors, or provide misinformation, these inaccuracies make them unfit as independent tools. 

Although medical accuracy is paramount, LLMs’ responses must be readable to successfully serve their purpose as patient resources. The average adult in the United States reads at approximately a 7th-grade reading level [33]. Additionally, one study identified that in plastic surgery, 50.2% of patients had an education level lower than high school and that 48% of attendings, residents, and PAs said it was challenging to make patients follow postoperative instructions [34]. Moreover, patient materials in plastic surgery are often above the recommended average reading level of 6th to 8th grade and may be too difficult for the average patient [34,35,36,37]. 

Vallurupalli et al. [37] successfully used ChatGPT-3.5 to significantly simplify traditional patient education materials for craniofacial surgery by 3 FKGL points. Interestingly, in our study, ChatGPT-3.5 and 4’s average FKGL scores were far superior to the recommended reading level. This was consistent with the results of Momenaei et al. [38], where ChatGPT-4’s FKGL and FRE scores averaged at 14.3 and 31.6, respectively. Conversely, Gemini proved to be significantly superior in terms of readability, with responses at an average reading level of 10th grade and an average FRE score 10 points higher than that of the other models. Al-Sharif et al. [28] identified similar results, with GPT having a higher analytical reading inventory (ARI) score than BARD, indicating that a higher level of education was required to understand its responses. Perhaps providing tailored prompts requesting a specific reading level might consistently improve the readability of the answers. However, this might risk oversimplifying the responses and missing essential information. 

Gemini also outperformed both ChatGPT models in providing understandable answers. This was because the PEMAT contemplates the use of visual aids as part of its score. Gemini created images for three responses, two of which helped improve understandability. Additionally, in a question about breast augmentation complications, it provided a link to the FDA information website. While ChatGPT-4 also has the ability to create images due to its integration with DALL-E, it only does so when specifically asked to. The ability to think when it would be appropriate to include images for an explanation instead of waiting to be asked to is what determined Gemini’s superiority. Nevertheless, both GPT models scored above 85%, proving that they provide understandable answers. Similar to readability, tailoring the prompt so that it provides helpful images may improve further ChatGPT-4’s understandability. 

Overall, the three models performed poorly in actionability, scoring less than 60% on average. This was a consequence of the format in which the models presented their responses. A minimum of two out of the seven questions were not applicable, and while most of the time they provided clear, broken down actions, they rarely provided tangible tools such as checklists. As visual aids were also part of the actionability section, both GPT models could only score as high as 80%. Although Gemini did provide images, they were not useful for inspiring actionability, hence not showing any difference between the models. 

## 5. Strengths and Limitations 

To our knowledge, this is the first study comparing the two versions of ChatGPT and Gemini in terms of providing postoperative care recommendations to patients who have undergone cosmetic plastic surgery procedures. However, our study has some limitations. First are the limited number of questions prompted per procedure. This limited the depth in which we could analyze the models’ capacity to provide accurate, readable, understandable, and actionable responses for any particular procedure, limiting their clinical applicability. Furthermore, our findings may not generalize to other LLMs or fields within or outside of plastic surgery and are limited by the current training data of the models. Further research into the newest, more potent versions of Gemini may be paramount, especially considering the current version’s superiority to ChatGPT models. Moreover, with additional research constraining the LLMs to provide good-quality information, either through RAG, functional tuning, prompt engineering, or parameterization, we can leverage language understanding but restrict the answers to good, accurate information. Notably, as LLMs evolve rapidly and constantly, our results will likely differ from those obtained in the near future. Nevertheless, a continuous evaluation of model performance is crucial at all stages of development. The limitations and weaknesses highlighted by our study can provide valuable insights to guide future development and practice, especially toward specialty-specific models.

The present study evaluated the LLMs’ responses based on their textual contents without considering the patient–physician interaction and feedback. The impact on patient understanding, satisfaction, subsequent behavior, and real-life clinical settings remains unexplored. Lastly, our study primarily focused on English-language responses, demanding further research on the performance across different languages and cultural contexts. 

## 6. Conclusions

Our study provides valuable insights into the efficacy of LLMs in delivering postoperative care recommendations to patients who have undergone cosmetic plastic surgery. Although ChatGPT-4 is the most effective and latest updated version of OpenAI, the publicly available 3.5 version and Google’s Gemini public version provided equally accurate medical advice. However, Gemini proved to be superior in providing understandable and more readable responses. While all three models demonstrated their potential as adjunctive tools in postoperative patient care, their shortcomings in providing actionable and concise guidance highlight the need for additional refinement and research to enable their evolution into comprehensive standalone resources. Further experimentation is necessary to evaluate the performance of a retrieval-augmented generation technique.

## Data Availability

The raw data supporting the conclusions of this article will be made available by the authors upon request.

## Supplementary Materials

The following supporting information can be downloaded at: https://www.mdpi.com/article/10.3390/healthcare12111083/s1, Supplementary File S1. Questions provided to LLMs; Supplementary File S2. LLMs Responses.
