# Supplementary material for: Artificial Intelligence in Postoperative Care: Assessing Large Language Models for Patient Recommendations in Plastic Surgery
Source: Healthcare (Basel). 2024 May 24;12(11):1083. doi: 10.3390/healthcare12111083 (PMC11171524; doi:10.3390/healthcare12111083)
Supplement: Supplementary file 1 [file healthcare-12-01083-s001.zip › Supplementary File S1. Questions provided to LLMs.pdf]

Supplementary S1. Questions provided to LLMs.

1. When am I going to see the final outcome of my liposuction?
2. Is it normal to have multiple bruises after a liposuction?
3. I just had a liposuction and I feel like I am gaining weight, is this normal?
4. When can I shower if I had a liposuction?
5. How soon can I return to my normal activities after a liposuction?
6. Is it normal to have smelly fluid coming out of the drains from my liposuction?
7. How long should I wear the compression garment after a liposuction?
8. How can I manage pain after breast augmentation?
9. How long do breast implants usually last?
10. Can I wear my normal bra after breast augmentation?
11. It's been 2 weeks since my breast augmentation surgery, can I start doing yoga?
12. Can I touch my breasts after having breast augmentation surgery?
13. Are there any complications I should be aware of if I had breast augmentation surgery 3 months ago?
14. Is it normal to feel abdominal numbness after a tummy tuck?
15. How many days should I stay in bed after an abdominoplasty?
16. I just had and abdominoplasty, when is the doctor going to take out my drains?
17. When can I go to the beach and sunbath after a tummy tuck?
18. Can I sleep over my belly if I had an abdominoplasty almost 3 weeks ago?
19. When can I start going to the gym if I had a tummy tuck?
20. I just had a breast lift 5 days ago and I am having a headache, is there any drug I should avoid?
21. I had a breast lift some years ago and I feel that my breast are starting to sag, is that something normal or should I go with my surgeon?
22. I had a breast lift 6 months ago and one of my nipples is completely asymmetric in relation to the other, should I go visit my surgeon?
23. What should I take if I just had a breast lift 3 days ago and I'm having pain? Please give examples
24. How long should I wear a support bra if I had a mastopexy?
25. Is there anything I can do to avoid stiffness after a breast lift?
26. What are the most common complications I should watch out for if I just had eyelid surgery?
27. What should I do if I am seeing things double after eyelid surgery?
28. I am an asian woman who had eyelid surgery and I feel my eyes seem uneven, why could this be?
29. I just had eyelid surgery, can I sleep normally?
30. How many times a day can I apply eyedrops if I had eyelid surgery?
31. What can I do to avoid or decrease edema after eyelid surgery?
32. Can I eat normally if I had eyelid surgery?
